# Sustainability of the Pacific Diabetes Today Coalitions

**Published:** 2009-09-15

**Authors:** Nia Aitaoto, JoAnn Tsark, Kathryn L. Braun

**Affiliations:** Papa Ola Lokahi; Imi Hale-Native Hawaiian Cancer Network, Papa Ola Lokahi, and Pacific Diabetes Education Program, Honolulu, Hawaii; Imi Hale-Native Hawaiian Cancer Network, Papa Ola Lokahi, and University of Hawaii, Department of Public Health Sciences, Honolulu, Hawaii

## Abstract

**Background:**

The prevalence of diabetes is unusually high among the indigenous peoples of Hawaii and the US-associated Pacific Islands. Although diabetes programs developed elsewhere have been tried in these Pacific Islander communities, they have not been sustained. Research suggests that program sustainability is enhanced by the presence of a champion, the fit of the program in an organization, and assistance from stakeholders.

**Context:**

In 1998, the Pacific Diabetes Today Resource Center, funded by the Centers for Disease Control and Prevention, applied a community-empowerment approach to diabetes programming, providing training and technical assistance to coalitions in 11 US-associated Pacific Islands communities. When funding ended in 2004, many of the diabetes programs continued. In 2008, we revisited the 11 communities to examine the vitality of their diabetes coalitions and factors that were known to sustain the programs.

**Methods:**

We interviewed coalition representatives in each of the 11 communities about diabetes-related programming developed from 1999 through 2003 and factors influencing sustainability of diabetes-related activities.

**Consequences:**

Coalitions that continued the diabetes programming they developed for or adapted to their communities had community leaders (or champions), found supportive organizational homes for the programs, and had assistance. Four case studies show how these factors affected successful coalitions.

**Interpretation:**

Freedom to adapt programs to new cultural contexts was a key factor in sustaining diabetes programs in the region.

## Background

The prevalence of diabetes is unusually high among the indigenous peoples of Hawaii and the US-associated Pacific Islands (USAPI). Traditional Native Hawaiian and Pacific Islander lifestyles were active, and diets consisted primarily of low-fat, high-fiber foods from the land and sea (1). Today, most islanders have sedentary lifestyles, and their diets are high in calories, salt, fat, and refined foods (2). As the prevalence of chronic diseases, especially diabetes, has risen, island governments have spent increasingly larger portions of their health budgets on secondary and tertiary care. However, resources for chronic disease prevention and control have been limited (3).

Although diabetes programs developed elsewhere have been tried in Pacific Islander communities, they have not been sustained because of lack of long-term funding or lack of program fit, meaning that the imported programs were not appropriate to the culture or environments of these Pacific Islands (4). For example, a year before Pacific Diabetes Today Resource Center (PDTRC) training, 1 of our sites had piloted a diabetes support group following a model from a Native American community. The model proved inappropriate for cultural reasons: 1) the men and women did not want to meet together in a mixed group, 2) the time allotted for meetings was too short for meaningful interaction and relationship development, 3) the nurse was much younger than the patients, and 4) food was not provided at meetings. Through its Diabetes Today initiative, the Centers for Disease Control and Prevention (CDC) funded resource centers to train communities in coalition building and program planning skills so that communities could develop diabetes programs that were appropriate for the local culture.

From 1999 through 2003, the PDTRC provided training and technical assistance to 11 communities in Hawaii and the Pacific, including Kauai County in the state of Hawaii, American Samoa, the Commonwealth of the Northern Mariana Islands (CNMI), the 4 culturally distinct states — Chuuk, Kosrae, Pohnpei, and Yap — of the Federated States of Micronesia (FSM), Guam, Palau, and 2 communities — Majuro and Ebeye — in the Republic of the Marshall Islands (RMI) (4,5). All communities developed diabetes initiatives such as community outreach to increase screening, diabetes prevention education campaigns, diabetes trainings for local health care providers, and fundraising activities to purchase and provide medical supplies.

But were they sustained after PDTRC closed in 2004? Research suggests that program sustainability is enhanced by the support of a respected local leader (a champion), the fit of the program within a sponsoring organization, the ability to shape or modify the program, the perceived benefits of the program, and assistance from stakeholders (6). The purpose of this study was to revisit the 11 coalitions in 2008 to examine whether known sustainability factors contributed to the vitality of diabetes programming developed with the help of PDTRC.

## Context

Hawaii and the 6 US-associated Pacific Islands jurisdictions have unique and distinct cultures and languages. But these islands share a history of occupation by the United States. The United States has used its Pacific possessions for military purposes, including nuclear weapons testing (3,5,7). Relationships with the United States have continued into the 21st century. Hawaii is a now a state, American Samoa (7) and Guam (8) are unincorporated US territories, and the CNMI is a US commonwealth (9). The FSM (10), the RMI (11), and Palau (12) are independent nations with compacts of free association with the United States, collectively referred to as Freely Associated States. All are eligible for health-related US government funding.

Native Hawaiians have the highest diabetes mortality rates of major ethnic groups in Hawaii, approximately 8% (13). The World Health Organization and diabetes specialists in the region note a high prevalence of diabetes in all US-associated Pacific Island jurisdictions (14). Estimates suggest that 94% of American Samoans are overweight or obese and 47% have diabetes. In the RMI, estimated diabetes prevalence is 30%, and amputation rates increased by 28% from 2000 to 2001. In the CNMI, a survey of 10th-grade students found that 78% of students had family members with diabetes and 64% of the students had 3 or more risk factors (eg, family history, high body mass index [BMI], elevated cholesterol, high blood pressure, tobacco use). In Guam, diabetes prevalence doubled from 5% in 1996 to 10% in 2003. A door-to-door survey in Palau yielded a diabetes prevalence of 14% in the group aged 50 to 64 years and 23.5% in the group aged 65 or older (Pacific Diabetes Education Program, Pacific Advisory Council meeting minutes 2007, unpublished data).

A 1998 community assessment in our 11 communities identified several contributors to the high diabetes prevalence, including lack of knowledge about health and diabetes, poor lifestyle behaviors, and lack of health services and healthy lifestyle infrastructures. Informants reported that many diabetes programs tried in Hawaii and the Pacific were developed by outsiders unfamiliar with Pacific cultures, infrastructures, and local politics. These programs were difficult to implement because of cultural mismatch and difficult to maintain because funding was short-term (1-3 years). Thus, by the time people became familiar with the program, it was discontinued, and this pattern repeated itself with each new funder (5,15).

To respond to the need for community-driven programs and continuity, PDTRC was established in 1998 by Papa Ola Lokahi (a nonprofit organization dedicated to Native Hawaiian health and well-being) to support community-based coalitions to address diabetes through capacity building. Funded by CDC, PDTRC trained community members in 11 communities in the region to conduct a community assessment and to plan, implement, and evaluate diabetes prevention or control programs within their communities (5). In the first year of this 5-year project, we listened to community sentiment through semistructured discussion groups, convened a Pacific-wide advisory council, and gathered data from informants throughout the region that led to the tailoring of CDC's Diabetes Today curriculum for the Pacific (15). The PDTRC curriculum was pretested in year 2 and further modified on the basis of participant feedback. In years 3 through 5, we used the curriculum to train 11 community groups and provided technical assistance to facilitate group development, planning, resource identification, and project implementation (4,5).

PDTRC's work was guided by the principles of community building and the goal of empowering coalitions to take action regarding diabetes. Activities were aimed at strengthening individual competence and community capacity to identify and resolve problems (16,17). For example, we engaged Native Hawaiians and Pacific Islanders in identifying community health concerns, defining program priorities, and developing culturally appropriate processes and products. In accordance with capacity-building principles, we employed culturally appropriate strategies to gain access to communities, transfer knowledge and skills to people, strengthen community coalitions, and provide technical assistance (5,15,18). For example, to gain access to 1 of our Pacific communities, our staff met with the local health department, which formed a team to present PDTRC to the island-wide council of chiefs. After the council gave permission for the team to enter the village, the team met with local leaders. The team followed local protocols, such as asking for an invitation before entry, exchanging cultural gifts with the leaders, listening to elders in developing training agenda, and respecting cultural protocols related to inclusion, prayer, food, and so forth. When CDC's Diabetes Today funding ended in 2003, all 11 coalitions were implementing successful diabetes activities.

PDTRC included discussion about sustainability in all of the advisory council meetings so that everyone understood that funding could not be guaranteed beyond the contract period. Thus, the advisory council understood that PDTRC funding was to be used for community capacity building, rather than programming. After PDTRC funding ended, we wondered how well the 11 coalitions would sustain themselves and their respective diabetes activities. In 2003, we hypothesized that coalitions needed 3 factors to continue offering programs adapted to their communities: a supportive host agency for the coalition-developed program, a leader or champion for the program, and continued access to technical assistance (18). In a literature review on program sustainability, Scheirer identified a similar set of sustainability factors: a champion, program-organization fit, ability to adapt the program, perceived program benefits, and assistance (6).

## Methods

Although PDTRC funding ended in 2004, Papa Ola Lokahi was awarded another CDC grant in 2005. We called this 5-year National Diabetes Education Program cooperative agreement the Pacific Diabetes Education Program (PDEP). This initiative helped us maintain contact with the 11 PDTRC groups, and we freely provided technical assistance when asked. In addition, each group was invited to participate in PDEP to receive support for developing and distributing culturally and linguistically appropriate diabetes educational materials (PDEP, Pacific Advisory Council meeting minutes 2007, unpublished data).

In 2008, we revisited the 11 community coalitions trained by PDTRC and interviewed our original in-country sponsors of the PDTRC training. The interviews were conducted in person by the first author (NA), who was lead trainer for both PDTRC and PDEP. Interview questions were broad and open-ended: 1) Is your coalition actively engaged in diabetes prevention and control activities? If not, when was the last time you implemented activities? 2) Does your coalition have a champion? 3) Is your coalition or diabetes program housed in a supportive agency committed to your mission? 4) Does your coalition have access to resources and technical assistance? Interview notes were transcribed, and a copy was sent by e-mail to the PDTRC/PDEP contacts for review. Clarification was done by e-mail and phone.

From these data, we learned that 9 of the 11 coalitions were still actively conducting diabetes-related programs. To further explore elements influencing success, we examine the impact of sustainability factors on 4 of the 9 coalitions. These 4 represent diverse host agencies within which they operate. Specifically, the Guam Diabetes Association is a nongovernment organization (NGO) dedicated to diabetes prevention and control. The Chuuk Women's Council is an NGO that did not focus on health before its affiliation with PDTRC. Both the Kosrae Diabetes Today and the Kauai Diabetes Today Coalition were founded by people who attended PDTRC training (initially sponsored by their health department). We also feature these 4 sites because they represent various political affiliations with the United States. Chuuk and Kosrae are Freely Associated States, Guam is a US territory, and Kauai is in Hawaii, a US state.

For each of the selected coalitions, we interviewed the original PDTRC contact, along with 3 additional informants: the identified "champion" for diabetes, a health care provider affiliated with the coalition, and a person with diabetes involved with the coalition's activities. We asked the following questions: 1) Tell us about your coalition since the PDTRC training occurred; 2) Why do you think your coalition is still going on, when many groups disband after a few years? What makes your group different?; 3) What diabetes prevention and control activities does your coalition do? How do you keep these programs going? What has helped you?; 4) What does your group have planned for next year?; and 5) What else would you like to share about your coalition or program? Answers were solicited from the 4 representatives in a conference call. Drafts of the individual case studies were prepared and sent to the respective interviewees for review, clarification, and approval.

Of the 23 participants in this study, the average age was 53 years; 13 (57%) were female, and 11 (48%) had diabetes. Each received a small (US $10) gift. In May 2008, the findings were e-mailed to all 23 participants and presented at the annual PDEP meeting. Eighteen of the 23 interviewees and 9 other PDEP members attended this meeting.

## Consequences

Of the 11 coalitions, 9 were still active in 2008. They all had an identified champion, were attached to solid and supportive organizations, and continued to seek and receive technical assistance from contacts made through affiliations with PDTRC and PDEP (Table). Interestingly, the 2 inactive coalitions were the same 2 coalitions that elected not to participate in our National Diabetes Education Program grant — the American Samoa Diabetes Coalition and the Yap Diabetes Group. We learned that the champion of the American Samoan group passed away in 2003, and the group was still reorganizing itself in 2007. The champion for the Yap group, a local primary care physician, passed away in 2007. The 4 case studies that follow examine the operational sustainability factors in the respective communities.

## Four Case Studies

### Chuuk Women's Council

The Chuuk Women's Council (CWC) is an NGO in Chuuk State, FSM. It was chartered in 1980 to help women become more productive and self-sufficient. CWC has groups in the 40 inhabited islands within Chuuk State; membership ranges from 40 to 120 women per island.

When PDTRC entered Chuuk, the community identified CWC as a potential host agency for the training. Although CWC's focus was not health, PDTRC saw benefits in working with an existing, well-networked organization. PDTRC approached 2 CWC leaders to consider including health and diabetes in their mission. CWC accepted the offer because diabetes was a recognized health problem in the community and among its members. PDTRC training was held in 2002, and CWC subsequently embarked on diabetes programming. The 2 CWC champions are well-known community, church, and business leaders; one has diabetes, and the other has family members with diabetes. They continue to promote diabetes prevention, nutrition activities, and physical activity.

CWC also has implemented numerous faith-based diabetes initiatives and sponsors diabetes screenings in the communities. CWC members continue to provide support to people with diabetes through home and hospital visits. CWC has since expanded its health focus beyond diabetes, providing community education on sexually transmitted diseases, tuberculosis, and breast and cervical cancers. CWC continues to receive support from the local health department, other local health organizations, regional health associations, and PDEP.

### Kosrae Diabetes Today Coalition

In 2000, there were no community groups in Kosrae State of FSM focusing on diabetes. PDTRC partnered with Kosrae's health department to coordinate PDTRC training. After the training, PDTRC participants formed the Kosrae Diabetes Today Coalition (Kosrae DTC) to increase diabetes awareness and prevent the onset of diabetes and its complications. The founding membership included people with diabetes, policy makers, church leaders, and health care providers. The champion for the Kosrae DTC is a political and church leader whose energy and enthusiasm have not waned since the PDTRC training. He and most of the founding members are still engaged in leading the coalition and sponsoring diabetes-related activities.

One year after training, the Kosrae DTC became independently chartered as an NGO. A major focus of Kosrae DTC has been physical activity. The group has successfully influenced the mayors and traditional leaders to improve street lights and sidewalks to encourage physical activity, equipped villages with physical activity equipment such as volleyballs and volleyball nets, sponsored island-wide sporting events, and effectively lobbied the government (Kosrae's largest employer) to allocate part of the workday to exercise. Volleyball games are under way regularly in all 4 municipalities. One primary care physician has used a volleyball game site to promote weight loss by helping players track their weight loss over time. Kosrae DTC receives funding and technical assistance from the local department of health, churches, and women's group, and also from PDEP.

### Guam Diabetes Association

The Guam Diabetes Association (GDA) was the only existing diabetes coalition in the Pacific (outside of Hawaii) before the PDTRC training. GDA's mission is to help people with diabetes stay healthy. Its champions, the president of GDA (who has diabetes) and his wife, are founding members. Under their leadership, the association has grown to more than 200 members since its inception in 1982. PDTRC trained GDA leaders and members on program assessment, planning, and evaluation in 2002. GDA used the PDTRC training to plan the expansion of their community diabetes education and screening outreach to more rural areas. Following training, GDA successfully established diabetes coalitions in other villages in Guam. It expanded its annual Diabetes Conference to become the largest in the Western Pacific, attracting an average of 500 attendees. GDA's annual 5K run/walk averages 4,000 participants. The organization continues to participate in PDEP as well.

### Kauai Diabetes Today Coalition

On Kauai, a county in Hawaii, PDTRC partnered with the health department to offer training in 2002. The Kauai Diabetes Today Coalition (KDTC) was established by the trainees. Within a year, the group became a 501c3 organization with a membership of 40 people. The group's 2 champions are civic leaders; 1 has diabetes, and the other has family members with diabetes.

During the PDTRC training, the Kauai group identified several community needs, including the need to provide supplies such as glucometers and blood glucose test strips to uninsured and underinsured residents. KDTC subsequently held several fund-raising events, including a Valentine's Day Sweetheart Ball, for diabetes supplies and other KDTC activities. The Sweetheart Ball has since become a popular annual event, raising about $10,000 a year. KDTC also hosts an annual healthy picnic and participates in multiple community events, including most health fairs on Kauai. The group receives technical assistance and support from the local health department and PDEP.

## Interpretation

Our findings confirm the importance of the following key factors to sustaining programs that are developed or adapted by community coalitions: namely, a champion, a supportive host agency, and access to technical assistance and resources (5,6,18).

### Champion

Each of the 11 coalitions stressed the importance of having a champion. The 2 inactive coalitions gave "loss of champion"  as the key reason for their inactivity. The 9 active coalitions all identified champions, who included people with diabetes, family members of people with diabetes, health care providers, politicians, pastors, and traditional leaders.

### Host agency

To sustain activities, a host agency must be supportive by providing a place to meet, office space, and logistical support. It needs to manage funds raised through grants, contracts, and activities. In our experience, it does not matter if the host agency already existed (like CWC and GDA), was founded by the coalition that formed out of the initial PDTRC training (like the Kosrae and Kauai Diabetes Today organizations), or was a health department (like the RMI and Palau). The important thing is that the diabetes programs fit the mission of the host agency, which will justify continued support.

Among the 4 coalitions featured in the case studies, 7 leaders were recognized, some of whom were recognized as "champions."  All of the identified leaders initially served as the coordinators of the PDTRC training, which they also attended. As staff of a host agency, they continue to support and coordinate activities and report spending 60 to 160 hours per month (some of which are volunteer hours) on diabetes activities, such as recruiting new volunteers, encouraging members, providing individual and group education, coordinating big events, educating policy makers, and fundraising.

### Access to technical assistance and resources

All 11 coalition interviewees emphasized the importance of access to technical assistance and resources. In addition to receiving technical assistance from their host agencies, many coalitions cultivated and received resources from other local organizations, including churches, women's groups, and businesses. Types of resources included monetary support, incentives and prizes, volunteer time, and free advertisements. The 9 active coalitions also participated in PDEP, through which they accessed technical assistance. Because the 2 currently inactive coalitions did not participate in PDEP, we conclude that access to continuous technical assistance is a critical factor for sustainability.

### Other sustainability factors

Our findings confirmed additional factors that Scheirer identified in her literature review of program sustainability (6). The goal of PDTRC was to help coalitions develop and tailor programs that fit their communities because these would result in more tangible benefits and would be easier to sustain over time than programs developed elsewhere (6). Through PDTRC, all 11 coalitions gained skills to develop and implement their own programs (4,5). They had complete control over their programs and modified them at will. PDTRC (and later PDEP) helped coalitions customize their plans instead of forcing packaged programs on them. Successful coalitions also realized numerous benefits. Primarily, coalition activities increased awareness of diabetes and expanded diabetes prevention and control activities in communities. Several coalitions also provided direct support to people with diabetes; for example, CWC provides spiritual and caregiving support to patients through home visits, and KDTC provides diabetes supplies to diabetes patients. Leaders and champions affiliated with active programs gained skills and enjoyed increased recognition within their communities.

## Conclusion

Our findings confirm those of Scheirer and others concerned with program sustainability (6). Because of the success of 9 of our initial 11 PDTRC projects, we are confident that the PDTRC approach (building community capacity through training and technical assistance to develop community-defined programs) provided a good base for developing and sustaining diabetes-related programming in the region. Local champions, supportive host agencies, and ongoing technical assistance are critical to long-term sustainability. Papa Ola Lokahi is currently applying this approach to develop and expand programming in cancer and tobacco control in Hawaii and the US-associated Pacific Islands.

## Figures and Tables

**Table.** Status Report and Inventory of Critical Factors for Coalitions Trained by the Pacific Diabetes Today Resource Center, 2008

PDTRC Coalition	PDEP Participant	Status	Critical Factors		
			Committed Host Agency	Champion	Access to Resources, Technical Assistance
Marshall Islands Diabetes Association (Majuro)	Yes	Active	Yes^a^	Yes	Yes
Ebeye Diabetes Association	Yes	Active	Yes^a^	Yes	Yes
Kosrae Diabetes Today	Yes	Active	Yes^b^	Yes	Yes
Chuuk Women's Association^c^,^d^	Yes	Active	Yes^e^	Yes	Yes
Guam Diabetes Association^d^	Yes	Active	Yes^b^	Yes	Yes
Commonwealth Diabetes Association (CNMI)	Yes	Active	Yes^b^	Yes	Yes
Pohnpei Lipaiere	Yes	Active	Yes^b^	Yes	Yes
UAK (Palau)	Yes	Active	Yes^a^	Yes	Yes
Kauai Diabetes Today	Yes	Active	Yes^b^	Yes	Yes
American Samoa Diabetes Association	No	Not Active	Yes^b^	No	Yes
Yap Diabetes Group	No	Not Active	No	No	No

Abbreviations: PDTRC, Pacific Diabetes Today Resource Center; PDEP, Pacific Diabetes Education Program; CNMI, Commonwealth of the Northern Mariana Islands; UAK, Ulkerreuil A Klengar.

a An advisory group affiliated with the health department.

b Independent nonprofit organization devoted entirely to diabetes prevention and control.

c In existence before PDTRC.

d Nonhealth focus before PDTRC.

e Independent nonprofit organization that offers diabetes programming among other things.
